# Medicalization of female genital cutting in Malaysia: A mixed methods study

**DOI:** 10.1371/journal.pmed.1003303

**Published:** 2020-10-27

**Authors:** Abdul Rashid, Yufu Iguchi, Siti Nur Afiqah

**Affiliations:** 1 RCSI and UCD Malaysia Campus, George Town, Penang, Malaysia; 2 Ritsumeikan Asia Pacific University, Beppu, Oita, Japan; 3 University Sains Malaysia, George Town, Penang, Malaysia; Ghent University, BELGIUM

## Abstract

**Background:**

Despite the clear stand taken by the United Nations (UN) and other international bodies in ensuring that female genital cutting (FGC) is not performed by health professionals, the rate of medicalization has not reduced. The current study aimed to determine the extent of medicalization of FGC among doctors in Malaysia, who the doctors were who practiced it, how and what was practiced, and the motivations for the practice.

**Methods and findings:**

This mixed method (qualitative and quantitative) study was conducted from 2018 to 2019 using a self-administered questionnaire among Muslim medical doctors from 2 main medical associations with a large number of Muslim members from all over Malaysia who attended their annual conference. For those doctors who did not attend the conference, the questionnaire was posted to them. Association A had 510 members, 64 male Muslim doctors and 333 female Muslim doctors. Association B only had Muslim doctors; 3,088 were female, and 1,323 were male. In total, 894 questionnaires were distributed either by hand or by post, and 366 completed questionnaires were received back. For the qualitative part of the study, a snowball sampling method was used, and 24 in-depth interviews were conducted using a semi-structured questionnaire, until data reached saturation. Quantitative data were analysed using SPSS version 18 (IBM, Armonk, NY). A chi-squared test and binary logistic regression were performed. The qualitative data were transcribed manually, organized, coded, and recoded using NVivo version 12. The clustered codes were elicited as common themes. Most of the respondents were women, had medical degrees from Malaysia, and had a postgraduate degree in Family Medicine. The median age was 42. Most were working with the Ministry of Health (MoH) Malaysia, and in a clinic located in an urban location. The prevalence of Muslim doctors practising FGC was 20.5% (95% CI 16.6–24.9). The main reason cited for practising FGC was religious obligation. Qualitative findings too showed that religion was a strong motivating factor for the practice and its continuation, besides culture and harm reduction. Although most Muslim doctors performed type IV FGC, there were a substantial number performing type I. Respondents who were women (adjusted odds ratio [aOR] 4.4, 95% CI 1.9–10.0. *P* ≤ 0.001), who owned a clinic (aOR 30.7, 95% CI 12.0–78.4. *P* ≤ 0.001) or jointly owned a clinic (aOR 7.61, 95% CI 3.2–18.1. *P* ≤ 0.001), who thought that FGC was legal in Malaysia (aOR 2.09, 95% CI 1.02–4.3. *P* = 0.04), and who were encouraged in religion (aOR 2.25, 95% CI 3.2–18.1. *P* = 0.036) and thought that FGC should continue (aOR 3.54, 95% CI 1.25–10.04. *P* = 0.017) were more likely to practice FGC. The main limitations of the study were the small sample size and low response rate.

**Conclusions:**

In this study, we found that many of the Muslim doctors were unaware of the legal and international stand against FGC, and many wanted the practice to continue. It is a concern that type IV FGC carried out by traditional midwives may be supplanted and exacerbated by type I FGC performed by doctors, calling for strong and urgent action by the Malaysian medical authorities.

## Abstract 2

Abdul Rashid and co-workers study Malaysian doctors' positions on and experience of female genital cutting.

## Author summary

**Why was this study done?:**

- The United Nations (UN) and other international bodies strongly oppose female genital cutting (FGC) performed by doctors.
- There are reports that more doctors are performing FGC in many countries, but nothing is known about the practice among doctors in Malaysia.
- The current study was done to determine the extent of the practice of FGC among doctors in Malaysia, who the doctors were who practiced FGC, how and what was practiced, and the motivations for the practice.

**What did the researchers do and find?:**

- We recruited 366 doctors from 2 medical associations with large number of Muslim members from 2018 to 2019 to participate by answering a self-administered questionnaire.
- In addition, we conducted in-depth interviews with 24 doctors to provide clarity on the questions answered in the questionnaires.
- About 20% of the doctors performed FGC. Women doctors, doctors who owned or jointly owned a clinic, those who thought FGC was legal, and those who were encouraged in Islam and thought FGC should continue were more likely to perform FGC.
- Most doctors performed FGC on the skin over the clitoris, but some cut part of the clitoris.
- The main reasons cited for the practice were religious obligation, culture, and to prevent parents from seeking traditional midwives to conduct FGC.

**What do these findings mean?:**

- Some doctors were beginning to conduct more harmful forms of FGC that were never previously performed by the traditional midwives in Malaysia.
- Medical authorities in Malaysia should ban the practice of FGC by doctors and other health professionals.

## Background

The term female genital cutting (FGC; also known as female genital mutilation) refers to all procedures involving partial or total removal of the external female genitalia, or any other injury to the female genital organ for nonmedical reasons [1, 2]. There are several types defined by the World Health Organization (WHO) [1] that are practiced among countries.

Type I: Partial or total removal of the clitoris and/or the prepuce (clitoridectomy)

Type II: Partial or total removal of the clitoris and the labia minora, with or without excision of the labia majora (excision)

Type III: Narrowing of the vaginal orifice with creation of a covering seal by cutting and apposition of the labia minora and/or the labia majora, with or without excision of the clitoris (infibulation)

Type IV: Unclassified; all other harmful procedures to the female genitalia for nonmedical purposes, e.g., pricking, piercing, incising, scraping, and cauterization

FGC is controversial and has been labelled as a harmful traditional practice. The medical fraternity has described the procedure as harmful both physically and mentally, lawyers condemn it because it violates the human rights of children, and feminists argue that these procedures are a manifestation of gender inequality and detrimental to women’s health [3].

There are numerous reported acute or chronic health effects resulting from FGC, which can be classified into short- and long-term physical [4] and psychological and social [5] problems. Most physical and mental health issues are related to types I, II, and III [6] and the skill of the practitioner and the condition of the instruments [7]. FGC is practiced in numerous African countries and in some nations in Asia and the Middle East, and because of migration, this practice is now even reported in countries where historically FGC was never practiced [8]. It has been estimated that, worldwide, approximately 3.6 million girls are cut each year [2] and more than 200 million girls and women have undergone some form of FGC [9]. It is estimated that by 2050, the number of girls undergoing FGC will rise although the percentage of girls undergoing FGC may decrease [2]. Because of the strong cultural and religious values and belief surrounding FGC [1, 10–12], the decline in prevalence is slow despite decades of campaigning and even criminalizing FGC [13]. FGC occurs across socioeconomic classes and among different ethnic groups, cultures, and religions [14]; however, many of the communities that practice it are Muslims in spite of FGC not being mentioned in the Quran and opposition to this practice by some religious personalities [15].

In 2012, the United Nations (UN) General Assembly adopted a resolution calling for global efforts to end the practice. A wide range of intervention strategies have been implemented with the goal of accelerating abandonment of FGC, including reducing the extent of cutting, changing the age at which FGC is carried out, and promoting its medicalization [13]. But evidence is lacking that medicalization is the first step to the elimination of the practice [13, 16, 17]. WHO defines medicalization as the “situation in which FGC is practiced by any category of healthcare provider, whether in a public or private clinic, at home, or elsewhere” [1, 18]. This definition was adopted by the UN in a joint policy statement of WHO/United Nations Children’s Fund (UNICEF)/United Nations Population Fund (UNFPA) issued in 1997 [19] and reaffirmed by 10 UN interagency statements in 2008 [20].

Traditionally, most of those who cut are traditional healers, who have no medical training and perform without any anaesthesia nor sterilization [4]. Now, more parents are choosing to have their daughters undergo the procedure by healthcare providers preferably in a clinic to minimize pain and complications [21]. This trend in the medicalization of FGC is a serious global concern [18].

It is estimated that, worldwide, more than 18% of FGC procedures are carried out by healthcare workers, which includes nurses, trained midwives, and other healthcare professionals. The rates vary between 1% and 74% among countries [16]. Most of the medicalization is reported in Africa. The involvement of healthcare providers has been labelled as unprofessional and a violation of the medical code of ethics and is even illegal in some countries. Medicalization creates a false impression that the procedure is good for health or harmless and potentially creates a sense of legitimacy for the practice [16]

The increase in the demand for healthcare providers to perform FGC is postulated to be due to the increase in awareness of the community to the harmful health consequences of the practice if performed by traditional practitioners using unsterilized instruments and who do not have knowledge about the anatomy and physiology of the human body and principles of infection prevention nor the training required to treat the consequences [1]. The common reasons cited by doctors who practice FGC are as follows.

### 1. Harm reduction [1, 12, 16, 17]

The doctors believe that they are preventing the risks associated with FGC performed by traditional practitioners and that if they do not provide the service, the community will revert to traditional practitioners. The harm reduction argument has even been supported by doctors from countries in which FGC is not a “social norm” such as Belgium and the United States and even by some nongovernmental organisations (NGOs) [12, 22, 23]. Medicalization as a harm reduction strategy has been shown to be effective in locations where more serious forms of FGC is practiced [16, 24]. But using harm reduction as an excuse to practice FGC is controversial. The goal of harm reduction is to reduce the health consequences of various behaviours for both the individual and the community in which they live by offering a pragmatic and culturally acceptable set of alternatives [25]. Most harm reduction strategies are usually conducted among individuals who can give informed consent and involve strategies that are reversible. But because children are unable to give consent and FGC is not reversible, the principles of harm reduction do not apply to medicalization of FGC [23], and by promoting it as harmless and hygienic is construed as promoting medicalization.

### 2. Religion [1, 12, 16, 23] and support for the parents’ sociocultural beliefs [1, 12, 13, 16, 26]

Most healthcare providers who perform FGC are part of the FGC-practising community in which they serve and often have the same motivations as those requesting FGC.

### 3. Financial gains as a motivation for FGC [1, 5, 16, 23]

It is reported that health practitioners fear social sanctions if they do not practice FGC especially in rural communities, where members of the community may boycott their practice, resulting in lower patient numbers and reduced income [27].

The global commitment to eliminate all forms of FGC by 2030 is stated in target 5.3 of the global Sustainable Development Goals and the joint interagency Global Strategy to Stop Health-Care Providers from Performing FGC [18]. The World Health Assembly adopted a resolution that member states agreed to work on toward the elimination of FGC and toward ensuring that the procedure is not performed by health professionals. The World Medical Association along with the International Federation of Gynaecology and Obstetrics (FIGO), UN Treaty Monitoring Bodies, and numerous NGOs have condemned the medicalization of FGC and have called on countries to eliminate medicalization [1]. Despite this, FGC is increasingly performed by health professionals worldwide, particularly in Africa [16, 23, 28], but not much is known about the practice of FGC in countries in the South East Asian region where FGC is also conducted.

There is no nationally representative data on FGC in Malaysia [2]. There are few published articles on the practice of FGC, and besides a brief mention of medicalization, there are no data available on the medicalization of FGC in Malaysia. Malaysia is located in South East Asia with a population of about 26 million, 54.6% of whom are Malay Muslims—and of these, 27.1% are Malay Muslim women, according to the last census held in 2010 [29]. Malaysia is made up of 14 states and is divided into West and East Malaysia. Studies show that about 99% of Malay Muslim women have undergone FGC, mostly because they believe it is mandatory in Islam. FGC is usually performed by traditional midwives who practice type IV FGC. The midwives usually insist on a drop of blood as a requirement for the fulfilment of the practice. However, there is a trend in medicalization, wherein more younger women are cut by doctors and would prefer doctors to perform FGC on their daughters, mainly because of cleanliness and expertise. The community self-reported medicalization rate is about 28% [30–32]; however, there are no data on doctors practising FGC. Doctors in Malaysia are required to complete 4 years of compulsory service with the Ministry of Health (MoH) Malaysia before they are permitted to open their own practice either by owning or jointly owning a clinic or a group of clinics. Some doctors may choose not to own a practice but rather freelance between clinics acting as locum doctors. The law is silent on the practice of FGC in Malaysia, and the Malaysian Medical Council (MMC) has not stated its official stand on the practice of FGC among doctors. However, FGC is not a service offered by MoH Malaysia. The national religious department had issued a “fatwa” (a religious edict that is nonbinding) in 2009 that FGC is mandatory for Muslim women in Malaysia. However, religion is under the jurisdiction of each state rather than the federal government, and the states may issue their own fatwas.

The current study aimed to determine the extent of medicalization among doctors in Malaysia, the doctors who practice it, how and what is practiced, and the motivations for the practice.

## Methods

A brief protocol was prepared and is attached as Supporting Information (S1 Protocol).

### Study design

This was a mixed method (qualitative and quantitative) study conducted among Muslim medical practitioners registered as members in 2 major medical associations in Malaysia.

### Tool

A self-administered questionnaire was created for data collection (S1 Questionnaire). Doctors were given the questionnaire along with a client information sheet and a postage-paid envelope with the investigators’ address on it. Questions for the quantitative component of the study included age, sex, medical degree, year graduated, any postgraduate qualifications, and the location of clinic. Questions on practice included the following: years of practice, whether the respondent received training on FGC, where the respondent received FGC training (if applicable), number of FGCs performed, use of local anaesthesia, bleeding as a consequence of the procedure, complications, questions related to screening patients for bleeding disorders, questions related to infectious diseases and other health-related issues prior to the procedure, anatomical location of the procedure and what exactly was done, instruments used, age of patient, charges, reasons for performing FGC, and consent. For the qualitative part, in-depth interviews with doctors who practiced FGC were conducted using a semi-structured interview guide. The interviews focused on the reasons for practising and training received by the practitioners as well as the details of the procedure, which included how, where, and when FGC was conducted by the doctors.

### Population

The investigators had approached the largest medical association in Malaysia, which had approximately 11,500 members of which 2,905 were Muslims (1,426 male and 1,379 females), but this association did not approve the investigators’ request to help enrol their Muslim members into the study due to the sensitive nature of the research. Considering that FGC is related to Islam in Malaysia [32], the investigators enrolled Muslim doctors from 2 main medical associations in the country that had as members a large number of Muslim medical doctors from all over Malaysia. Association A had 510 members, of which there were 64 male and 333 female Muslim members. Association B had only Muslim members; 3,088 were female, and 1323 were male. With the help of these associations, the questionnaires were distributed during the annual conferences that were held by the associations and by posting the questionnaires to Muslim members who registered but did not attend. Because there were non-Muslim participants in one of the conferences, a member of the research team (who was given an opportunity to speak about the study during the conference before the questionnaires were distributed) had announced that only Muslim members were requested to fill in the questionnaires. Along with the envelope containing the questionnaire, a participant information sheet detailing the study objectives and rights of the participants, as well as the criteria for participation (which mentioned that Muslim doctors were eligible to participate), was included. Because there was a possibility, even if remote, that a doctor might be a member of the 2 associations and attend both conferences held within a month of each other, the participants were informed that only 1 questionnaire should be filled out if they should receive 2. The questionnaires were only posted to Muslim members of the association. In total, 894 questionnaires were distributed either by hand or by mail. In total, 300 questionnaires each were distributed at conference A and B; of these, 111 and 154 complete questionnaires were received back from the respective conferences. In total, 294 questionnaires were posted, and 101 completed questionnaires were received back. Those who were interested in participating in the in-depth interviews were invited to submit their name to the investigators during the conferences. These doctors further recommended names of colleagues who practiced FGC, who were then contacted and invited to participate in the study.

#### Sampling

The investigators were not able to find any published study related to FGC among medical doctors in Malaysia, and there are to date no official statistics relating to the practice of FGC among doctors. However, the investigators believed that a substantial number of doctors among the large population of Muslim doctors practiced FGC. Because the primary aim of the study was to describe prevalences, sample size was calculated for this, based on the Agresti-Coull binomial confidence interval. A sample size of 384 Muslim doctors would have allowed the study to determine the prevalence of those practising FGC with a confidence interval of ±5% with a prevalence of 50%.

For the qualitative part of the study, it was the intention of the investigators to interview the doctors until the data had reached saturation. To ensure that there was fair sampling of doctors interviewed, they were chosen from the northern east and west coast states as well as the central parts of peninsular Malaysia. No interviews were conducted among doctors in the south and in East Malaysia because of financial and time constraints. The doctors who were interviewed were not among those who participated in the quantitative part of the study.

#### Analysis

Quantitative data were analysed using SPSS version 18 and presented descriptively in tables and graphs. To estimate the factors associated with practising FGC, a chi-squared test was used for factors that included age, sex, country where graduated, years since graduation, postgraduate qualification, clinic ownership, clinic location, awareness of fatwa, belief about whether FGC is mandatory in Islam, belief about whether FGC is legal in Islam, belief about whether all Muslims perform FGC, reasons for doing FGC in Malaysia, belief about whether FGC should continue, and beliefs about who should perform FGC and why FGC should be performed in clinics. Factors from bi-variate associations with *p* < 0.2 were included in binary logistic regression. Data (S1 Data and S1 Data Dictionary) are available in Supporting Information. This study is reported as per the Strengthening the Reporting of Observational Studies in Epidemiology (STROBE) guidelines (S1 STROBE Checklist).

Qualitative data were collected using a semi-structured questionnaire, which was pilot tested on 3 doctors who were not included in the final data collection. Some minor additions were made to the semi-structured interviews as a result of the pilot tests. Additional questions were included concerning the role of midwives in the practice of FGC as the doctors in the pilot study volunteered information about the future of FGC and the role of midwives. After the doctors were contacted and appointments were made, the interviews were conducted by 2 investigators who were familiar with qualitative data collection, using face-to-face interviews at the doctor’s place of work. The interviews were conducted in English; however, the answers given were a mixture of English and Malay. Data were collected until saturation of information was achieved. Saturation of data was considered to have been achieved when no new information was availed from the respondents. Using the existing literature, grounded theory was used to analyse data. The data were transcribed manually, organized, coded, and recoded by one of the investigators using N vivo version 12. The clustered codes were elicited as common themes by the research team.

### Ethics

This study was ethically conducted, with all the participants providing written informed consent. The questionnaires, along with participant information sheet, were given to the participants, who were then required to sign the informed consent form before returning it to the investigators. For the qualitative interviews, the doctors were read out the information sheet, and they were required to sign the informed consent form before the start of the interview. The anonymity of the participants is assured; each participant was assigned a unique code by one of the investigators, who was also responsible for keying in the data. The questionnaires are stored in a locked cupboard in the office of one of the investigators, to which only he has access. The study received the ethical approval of Ritsumeikan Asia Pacific University Research Ethics Committee (2018–01).

## Results

### Baseline of respondents

In total, 366 completed questionnaires were received from participants from all states in Malaysia. As shown in Table 1, most of the respondents were women (73.8%), had medical degrees from Malaysia (69.7%), and had a postgraduate degree (61.5%) in family medicine. The median age was 42, and the range of years since graduating was 1 to 51 years (mean = 18.0). Most were working with MoH Malaysia (55.0%) and in a clinic located in an urban location (79.8%).

**Table 1:** Baseline information of respondents.

Variable	Number	Percentage
**Age (median, IQR)**	42 (34–52)	
**Sex**		
Men	96	26.2
Women	270	73.8
**Basic medical degree**		
Malaysia	255	69.7
Non-Islamic countries	77	21
Islamic countries	34	9.3
**Years since graduating**	1–51	
**Postgraduate qualification**		
Yes	225	61.5
No	141	38.5
**Types of postgraduate qualification**		
Postgraduate diploma	14	5.9
Internal medicine	16	6.7
Public health	13	5.4
Radiologist	1	0.4
Anaesthetist	2	0.8
Family medicine specialist	180	75.3
Master’s in science	6	2.5
Paediatrician	1	0.4
Psychiatrist	1	0.4
Surgeons	5	2.1
**Clinic ownership**		
MoH	203	55.5
Self	86	23.5
Joint	77	21.0
**Clinic location**		
Urban	292	79.8
Rural	74	20.2

**Abbreviation:** MoH, Ministry of Health

Table 2 shows the background of the doctors interviewed in depth; the mean age of the doctors was 49 years; most were women (95.8%), had graduated from a Malaysian institution (79.2%), were running their own clinic (79.1%), and did not possess a postgraduate degree (75.0%).

**Table 2:** Background of doctors interviewed in-depth.

Variables	*n*	%
**Age, years**	Mean = 49 (range 32–65)	
**Years since graduating**	Mean = 7.8 (range 7–34)	
**Sex**		
Women	23	95.8
Men	1	4.2
**Country of basic medical degree**		
Islamic country	3	12.5
Malaysia	19	79.2
Non-Islamic country	2	8.3
**Clinic**		
Joint	3	12.5
Locum	1	4.2
MoH Malaysia	1	4.2
Self	19	79.1
**Postgraduate degree**		
Yes	6	25.0
No	18	75.0

**Abbreviation:** MoH, Ministry of Health

### Details of FGC practice

Table 3 describes the details of the practice among the 75 (20.5%) Muslim respondents who reported that they practiced FGC. The average number of years the doctors had practiced FGC was 11.7 (range 1 to 33). Slightly more than half (53.3%) claimed to have received training on how to do FGC, mostly from colleagues (75%).

**Table 3:** Factors related to FGC practice.

Variable	Number	Percentage
**Practice FGC?**		
Yes	75	20.5
No	291	79.5
**Years practising**	1–33 (mean = 11.7)	
**Received training on FGC?**		
Yes	40	53.3
No	35	46.7
**If yes, where?**		
Medical school	1	2.5
Online	1	2.5
Colleagues	30	75.0
Self-taught	3	7.5
Religious personnel	4	10.0
Traditional midwives (“mak bidan”)	1	2.5
**How many FGCs performed per month?**	1–59 (SD 6.6)	
**Receive training?**		
Yes	8.8 (SD 9.7)	
No	4.2 (SD 6.8)	
**If received training, from whom**		
Medical school	6.0	
Online	4.0	
Colleagues	10.4 (SD 10.5)	
Religious personnel	4.3 (SD 5.1)	
Traditional midwives (mak bidan)	3.8 (SD 4.2)	
**Use local anaesthesia?**		
Yes	10	13.3
No	65	86.7
**Use of anaesthesia among those trained and not trained**		
Among those trained	6	60.0
Among those not trained	4	40.0
**Use of anaesthesia among those trained by different persons**		
Medical school	1	10.0
Online	1	10.0
Colleagues	4	40.0
Self-taught	1	10.0
Religious personnel	1	10.0
Traditional midwives (*Mak Bidan*)	2	20.0
**Any bleeding?**		
Yes	52	69.3
No	23	30.7
**How much blood?**		
A drop	51	98.1
Gauze full	1	1.3
More than a gauze	-	-
**Encountered any complication?**		
Yes	1	1.3
No	74	98.7
**Screen before conducting FGC?**		
Yes	28	37.3
No	47	62.7
**Screen for (multiple choice)**		
Infectious diseases	13	17.3
Bleeding disorders	15	20.0
Others	8	10.7
**Screening method (multiple choice)**		
History	20	26.7
Blood tests	-	-
Other	4	5.3
**What is done (multiple choice)**		
Excision of prepuce	7	9.3
Prick the prepuce	19	25.3
Nick the prepuce	22	29.3
Nick the tip of clitoris	18	24.0
Prick the clitoris	3	4.0
Others	6	8.0
**Instruments used (multiple choice)**		
Scissors	27	36.0
Surgical blade	11	14.7
Surgical needle	23	30.7
Other non-medical equipment	3	4.0
**What is applied post procedure?**		
Antiseptic	42	56.0
Antibiotic ointment	16	21.3
Nothing	16	21.3
Other	1	10.7
**Most common age of patient**		
0–3 months	20	26.7
4–6 months	23	30.7
7–12 months	24	32.0
>1 year	6	10.7
**Age procedure is suggested for?**		
0–3 months	23	30.7
4–6 months	26	34.7
7–12 months	20	26.7
>1 year	6	8.0
**Average charge?**	31.80 (range 0–100) (USD 1 = RM4)	
**Consent for FGC**		
Verbal	56	74.7
Written	7	9.3
None	12	16.0

**Abbreviations:** FGC, female genital cutting; USD, US dollar

Senior colleagues were mentioned most during the in-depths interviews as the persons whom they learned the procedure from, although some did mention traditional midwives as the source of training.

*“Learn from other doctors who did it*…*”* (Respondent 10)

*“…I learned from my friends and I even went to see a traditional healer to learn [to] do this……but I adjust a bit”* (Respondent 14)

On average, 6.6 FGCs were conducted (range 1 to 50) per month. The majority of respondents reported not using local anaesthesia (86.7%) and reported there was bleeding (69.3%), but only a drop of blood (98.1%). The overwhelming majority reported no complications (98.7%). Most (62.7%) did not screen patients for bleeding disorders or infectious diseases before commencing with FGC. Doctors who screened patients screened them for bleeding disorders (20.0%) and infectious diseases (17.3%), mostly by history taking (26.7%). Most doctors used instruments to nick (29.3%) and prick the prepuce of the clitoris (25.3%), most commonly using surgical scissors (36.0%), and they applied antiseptic (56.0%).

During in-depth interviews, pricking the prepuce of the clitoris was the common procedure described by the doctors.

*“We prick the prepuce*…*Just nick…just prick”* (Respondent 9)

*“Somebody teach [taught] me just to prick with the needle but I think it is not proper”* (Respondent 16)

However, a substantial number of respondents conducted their procedures on the clitoris itself.

*“There is nothing to be remove[d] except for the clitoris…you do like aaa…you remove small part but not actually small part…”* (Respondent 11)

*“…The tip of the clitoris*, *I cut it with the scissors”* (Respondent 20)

*“We cut*…*we try to get the small part of the clitoris*. *[asked again whether the clitoris is cut and not the prepuce over the clitoris]…No*, *no*…*ya [yes] clitoris is cut”* (Respondent 17)

*“…Then I cut a very small*, *very little piece of clitoris”* (Respondent 16)

The most common age performed on was 7 to 12 months (32.0%), but most doctors preferred performing the procedure on ages 4 to 6 months (34.7%). The average charge was RM31.80 (1 US dollar [USD] = RM 4), and most obtained verbal consent (74.7%).

### Factors associated with FGC practice

As shown in Table 4, age (*p* < 0.001), years since graduating (*p* < 0.001), not possessing postgraduate degree, clinic ownership (*p* < 0.001), and clinic location (*p* = 0.02) were significantly associated with the practice of FGC.

**Table 4:** Factors associated with the practice of FGC.

Variables	Practice FGC *n* (%)	Do not practice *n* (%)	Chi-square/*P* value	OR (95% CI)
**Age, years**				
<31	2 (4.4)	43 (95.6)	20.635/<0.001	Ref
31–40	17 (13.1)	113 (86.9)		8.92 (2.09–38.08)
>40	56 (29.3)	135 (70.7)		2.76 (1.52–5.01)
**Sex**				
Men	17 (17.7)	79 (82.3)	0.619/0.431	
Women	58 (21.5)	212 (78.5)		1.05 (0.94–1.17)
**Medical degree from**				
Non-Islamic country	15 (19.5)	62 (80.5)	0.061/0.805	1.01 (0.89–1.15)
Islamic country including Malaysia	60 (20.8)	229 (78.7)		
**Years since graduating**				
<11	14 (12.8)	95 (87.2)	24.626/<0.001	Ref
11–20	14 (12.5)	98 (87.5)		2.15 (0.93–4.97)
21–30	34 (37.4)	57 (62.6)		2.22 (0.96–5.13)
>30	13 (24.1)	41 (75.9)		0.53 (0.25–1.13)
**Postgraduate qualification**				
No	40 (31.5)	87 (68.5)	14.46/<0.001	2.14 (1.43–3.22)
Yes	35 (14.6)	204 (85.5)		Ref
**Clinic ownership**				
Self	46 (53.5)	40 (46.5)	86.172/<0.001	3.77 (1.91–7.42)
Joint	18 (23.4)	59 (76.6)		20.07 (9.57–42.1)
MoH	11 (5.4)	192 (94.6)		
**Clinic location**				
Rural	22 (29.7)	52 (70.3)	4.858/0.02	1.91 (1.07–3.41)
Urban	53 (18.2)	239 (81.8)		

**Abbreviations:** FGC, female genital cutting; MoH, Ministry of Health

### Reasons for FGC practice

The main reasons for doctors practising FGC were religion (76%) and health (16%), whereas the reasons cited for not practising FGC were not having any training to conduct FGC (87%), it being against their beliefs (6%), believing that FGC was against Islam (5%), and believing that FGC was against the law (2%).

In-depth interviews also showed religion as the most common reason cited by most doctors.

*“Being a Muslim*, *I believe it is a religious obligation*, *but I am not sure if it is wajib [mandatory]… but I believe in my religion and deep inside I believe we have to do it*. *Because there are certain things you cannot see*, *you cannot understand…you just follow”* (Respondent 21)

*“…Yes*, *basically because of religion*,*… you know there is [a] demand for it because of religion*, *I have to do it*, *I am a medical professional*, *but I still have to do and there is no other reason for it*. *If the patients want[s]*, *we just do it*…*”* (Respondent 6)

Culture was also mentioned often.

*“I think…ahhh…it is related to culture…because of the culture [reemphasises]*, *I think it is difficult to change…if the doctors stop doing and culture requires it done*, *where will they [parents] go and what will happen*?*”* (Respondent 12)

This is probably because most respondents related culture to religion.

*“I think probably for both [religion and culture]…well*, *people take culture and religion as the same……as equal*.*”* (Respondent 4)

Most of the doctors had their daughters undergo the procedure too, and religion was again the reason for doing it.

*“Yes it is wajib [mandatory]*,. . . . *I circumcised all my daughters”* (Respondent 22)

Health and medical indications were only mentioned in passing, but most mentions of them were related to the opportunity to examine for abnormalities.

*“[Medical benefit*?*] I can’t tell you…I don’t think I can find one*, *but maybe we can see abnormalities like…that is what I try to look for also…”*(Respondent 3)

### Knowledge related to FGC practice

As shown in Table 5, most respondents were unaware of the fatwa (edicts of Islamic law that are not legally binding) by the department of religious affairs of Malaysia (JAKIM) in 2009 that FGC is “wajib” (mandatory) for females apart from medical reasons (61.5%).

**Table 5:** Knowledge about reasons for FGC and performance of FGC.

Variables	Frequency *n* (%)	Practice FGC *n* (%)	Do not practice *n* (%)	Chi-square/*P* value	OR (95% CI)
**Know about JAKIM fatwa?**					
Yes	141 (38.5)	35 (24.8)	106 (75.2)	2.640/0.104	0.66 (0.39–1.09)
No	225 (61.5)	40 (17.8)	185 (82.2)		
**FGC mandatory in Islam?**					
Yes	136 (37.2)	26 (19.1)	110 (80.9)	5.862/0.053	0.40 (0.13–1.22)
No	184 (50.3)	45 (24.5)	139 (75.5)		0.29 (0.10–0.87)
Don’t know	46 (12.6)	4 (8.7)	42 (91.3)		Ref
**FGC legal in Malaysia?**					
Yes	250 (68.3)	45 (19.0)	205 (82.0)	5.264/0.072	1.94 (1.08–3.48)
No	39 (10.7)	7 (17.9)	32 (82.1)		1.94 (0.75–5.05)
Don’t know	77 (21.0)	23 (29.9)	54 (14.8)		Ref
**All Muslims perform FGC?**					
Yes	137 (37.4)	26 (19.0)	111 (81.0)	0.308/0.579	0.86 (0.51–1.46)
No/Don’t know	229 (62.6)	49 (21.4)	180 (78.6)		
**Reasons for doing FGC in Malaysia?**					
***Religion—compulsory***					
Yes	127 (34.7)	26 (20.5)	101 (79.5)	0.0/1.00	1.0 (0.59–1.71)
No	239 (65.3)	49 (20.5)	190 (79.5)		
***Religion—encouraged***					
Yes	172 (47.0)	45 (26.2)	127 (73.2)	6.405/0.011	1.15 (1.03–1.27)
No	194 (53.0)	30 (15.5)	164 (84.5)		
***Health reasons***					
Yes	124 (33.9)	17 (13.7)	107 (86.3)	4.295/0.021	1.98 (1.09–3.58)
No	238 (66.1)	58 (24.0)	180 (75.3)		
***Hygiene***					
Yes	127 (34.7)	16 (12.6)	111 (87.4)	7.437/0.006	2.27 (1.25–4.15)
No	239 (65.3)	59 (24.7)	180 (75.3)		
***Reduced libido***					
Yes	77 (21.0)	11 (14.3)	66 (85.7)	2.305/0.129	1.70 (0.85–3.42)
No	289 (79.0)	64 (22.1)	225 (77.9)		
***Increase sensitivity/libido***					
Yes	24 (6.6)	1 (4.2)	23 (95.8)	4.201/0.040	0.82 (0.84–0.90)
No	342 (93.4)	74 (21.6)	268 (79.4)		
***Helps during childbirth***					
Yes	11 (3.0)	2 (18.2)	9 (81.8)	0.37/0.847	1.16 (0.25–5.51)
No	355 (97.0)	73 (20.6)	282 (79.6)		
***Peer pressure***					
Yes	9 (2.5)	2 (22.2)	7 (77.8)	0.17/0.896	0.90 (0.18–4.42)
No	357 (97.5)	73 (20.4)	284 (79.6)		
***Family pressure***					
Yes	48 (13.1)	12 (25.0)	36 (75.0)	0.689/0.406	0.74 (0.37–1.51)
No	318 (86.9)	63 (19.8)	255 (80.2)		
***Other***					
Yes	10 (2.7)	4 (40.0)	291 (79.5)	2.401/0.21	0.37 (0.10–1.36)
No	356 (97.3)	71 (19.9)	285 (80.1)		
**Who performs FGC in Malaysia?**					
Traditional midwives	178 (48.6)	48 (27.0)	130 (73.0)	8.92/0.003	1.17 (1.05–1.31)
Trained midwives from MoH	90 (24.6)	16 (17.8)	74 (82.2)	0.54/0.46	1.26 (0.68–2.32)
Nurses	41 (11.2)	5 (12.2)	36 (87.8)	1.95/0.16	1.98 (0.75–5.22)
Medical doctors	256 (69.9)	66 (25.8)	190 (74.2)	14.63/<0.001	1.24 (1.13–1.36)
Medical specialist	138 (37.7)	28 (20.3)	110 (79.7)	0.006/0.941	1.02 (0.60–1.72)

**Abbreviations:** FGC, female genital cutting; MoH, Ministry of Health

Fatwa was also mentioned as a reason for practising and for continuing to practice FGC during in-depth interviews. But almost all the respondents interviewed were unaware of the details of the fatwa.

*“I think in 2009*, *if I am not mistaken*, *it is obligatory for us*, *if it brings harm then no need to do*,*…otherwise wajib [mandatory]”* (Respondent 22)

*“I have read somewhere*, *and I know there is a fatwa but I don’t know how to read [explain it] exactly to you*. *Fatwa said [stated] you have to do it for a Muslim*, *baby*, *women…”* (Respondent 21)

*“Yes…but I really forgot…but there is fatwa*, *I read it somewhere”* (Respondent 21)

Most doctors did not think FGC is mandatory in Islam, and they didn’t think all Muslims perform FGC (61.5%) or that it reduces libido.

*“Religiously yes*, *because there is [a] fatwa on it*…*the fatwa…supposedly circumcision reduces the libido but…I have my doubts [about FGC reducing libido]…yeaaa”* (Respondent 23)

But the majority assumed that it was legal (68.3%) in Malaysia.

During in-depth interviews, legality of the practice was an area that most of the respondents were unsure about, but most agreed that they would not conduct FGC if there were clear instructions from the medical council or if it was declared illegal.

*“…Against the law*?*…I hope no… I am not aware of it*, *[of] any law against it”* (Respondent 20)

*“Hmm…I think it’s legal…because there is no law stated that it is illegal…so far*, *they [the MMC] allow it*…*they didn’t say we cannot do…”*(Respondent 14)

*“It is not in black and white…basically…ermmm…I know it is a grey area*, *it is not documented*, *cannot do or must do…if there is any new regulation we just follow”* (Respondent 5)

*“I don’t know if [the] MMC allow us or not*, *but they never stopped us from practising it*. *Nothing that said [stated] we cannot practice it*. *If they stop us from doing it*, *we won’t do it*. *But there is no such act…I don’t know…whether legal or what*, *the mother brought the baby to me so the consent is there already…and I got their verbal consent*. *The parents brought*, *it is not that we go search for the patients*…*they come to us”* (Respondent 21)

Most were of the opinion that medical doctors commonly perform FGC (69.9%).

Regarding the differences in the reasons for FGC, religion (χ^2^ = 6.405, *p* = 0.01), health (χ^2^ = 4.295, *p* = 0.02), hygiene (χ^2^ = 7.437, *p* = 0.006), and increased libido (χ^2^ = 4.201, *p* = 0.04) were statistically significant; regarding differences in who usually performs FGC, traditional midwives (χ^2^ = 8.92, *p* = 0.003) and medical doctors (χ^2^ = 14.63, *p* < 0.001) were statistically significant.

### Attitudes toward continuation of FGC practice

As shown in Table 6, the majority of doctors were of the opinion that FGC should continue (85.4%) and that medical doctors should be the ones to conduct FGC (63.9%).

**Table 6:** Doctors’ opinions concerning the future of FGC.

Variables	Frequency *n* (%)	Practice FGC *n* (%)	Do not practice *n* (%)	Chi-square/*P* value	OR (95% CI)
**Should FGC continue?**					
Yes	276 (75.4)	68 (24.6)	208 (75.4)	11.841/0.001	1.22 (1.12–1.34)
No	90 (24.6)	7 (7.8)	83 (92.2)		
**Who should perform FGC?**					
Traditional midwives	10 (2.7)	2 (20.0)	8 (80.0)	6.318/0.177	1.46 (0.28–7.72)
Trained midwives from MoH	82 (22.4)	12 (14.6)	70 (85.4)		0.83 (0.11–6.26)
Nurses	13 (3.6)	3 (23.1)	10 (76.9)		0.79 (0.16–3.85)
Medical doctors	234 (63.9)	56 (23.9)	178 (76.1)		0.29 (3.13–25.92)
Medical specialist	27 (7.4)	2 (7.4)	25 (92.6)		Ref
**Why should FGC be performed in clinics?**					
No complications	47 (12.8)	15 (31.9)	32 (68.1)	4.319/0.038	0.49 (0.25–0.97)
Less complications	193 (52.7)	46 (23.8)	147 (76.2)	2.800/0.094	0.64 (0.38–1.08)
Hygiene	253 (69.1)	63 (24.9)	190 (75.1)	9.779/0.002	1.19 (1.08–1.31)
Comply with tradition and culture	37 (10.1)	12 (32.4)	25 (67.6)	3.602/0.058	1.19 (0.95–0.99)
Safety	247 (67.5)	59 (23.9)	188 (76.1)	5.374/0.020	1.14 (1.03–1.26)
Experience	158 (43.2)	39 (24.7)	119 (75.3)	2.998/0.083	1.09 (0.98–1.22)
Expertise	176 (48.1)	35 (19.9)	141 (80.1)	0.076/0.782	1.07 (0.65–1.79)

**Abbreviations:** FGC, female genital cutting; MoH, Ministry of Health

Religion was the main motivating factor behind the doctors’ belief that the practice should continue besides considering it to be a harmless procedure.

*“Yes*…*I would (continue to) do…in terms of religion I will do*. *Because there is not much harm*, *because it just a small prick and baby just cry like aah [expresses suggesting for a while] after we [are] done*, *it is okay*. . . .*Although I can’t think of any benefit for now*, *maybe when I am older*, *I will understand*. *Just like before this*, *I [didn’t] understand what fasting is for*, *what solat [prayer] is for*, *over time I understand [understood] it is good…*.*maybe it’s something that I have not discover[ed] yet*, *maybe my knowledge is still [gestures shallow]…If I don’t do*, *it is like a big sin*.*”* (Respondent 13)

There were doctors who suggested cosmetic reasons for the continuation of the practice.

*“It should be continue[d]…It should be because as I told you the shape of the labia is different from one person to another person*…*some babies just a bit exposed…(in others) there is a pouch but not as big…it depends [on the genitalia]*…*”* (Respondent 8)

During the in-depth interviews, no matter what the reasons were for practising, they all preferred the practice be conducted in a clinic by a health professional primarily as a harm reduction measure for the prevention of infections.

*“I think there is a need la [for doctors to perform]*…*because we do it in [a] sterile way compared to those ‘bidans’ [traditional midwives]…*. *Risk of infection is there and then risk of transferring infectious disease is there…*. *They are using blade…from what I understand*, *same blade from one person to another person…so I think the risk of infection is there”* (Respondent 2)

*“I don’t think they [traditional midwives] should continue doing*. *Because sometimes I hear from other’s experience…very bad practice*. *Very dirty…sometimes [they] use the same blade for one week…and the ‘kain’ [cloth] used to wrap [the tools] change colour [are stained]*. *…when we do*, *even if no SOP [standard operating procedure] we do in septic technique*.*”* (Respondent 13)

*“Some people [parents] who came here*, *they claimed [say] themselves they don’t want to go to the midwife because of hygienic reasons*, *the midwife[s] [are] already old and their eyesight not really clear [good]…*. *They [parents] think that it is not proper for the midwife to do [FGC] to their child”* (Respondent 17)

*“If we don’t do*, *they [parents] will do it outside*. *It is better to do in clinic*.*”* (Respondent 1)

Regarding the differences of opinion about whether the practice should continue (χ^2^ = 11.841, *p* = 0.001) and reasons why it should be performed in a clinic, there being no complications (χ^2^ = 4.319, *p* = 0.04), hygiene (χ^2^ = 9.779, *p* = 0.002), and safety (χ^2^ = 5.374, *p* = 0.02) were statistically significant.

**Fig 1:**
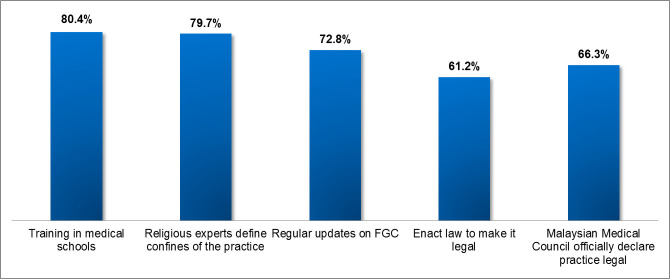
Doctors’ wish list for FGC. FGC, female genital cutting.

Fig 1 depicts the wish list of the doctors who want the practice to continue: wish that FGC be taught in medical schools (222), that religious experts define the confines of the practice (201), that there be regular updates on FGC (220), that the MMC officially declare FGC legal (183), and that law be enacted to make FGC legal (169).

**Fig 2:**
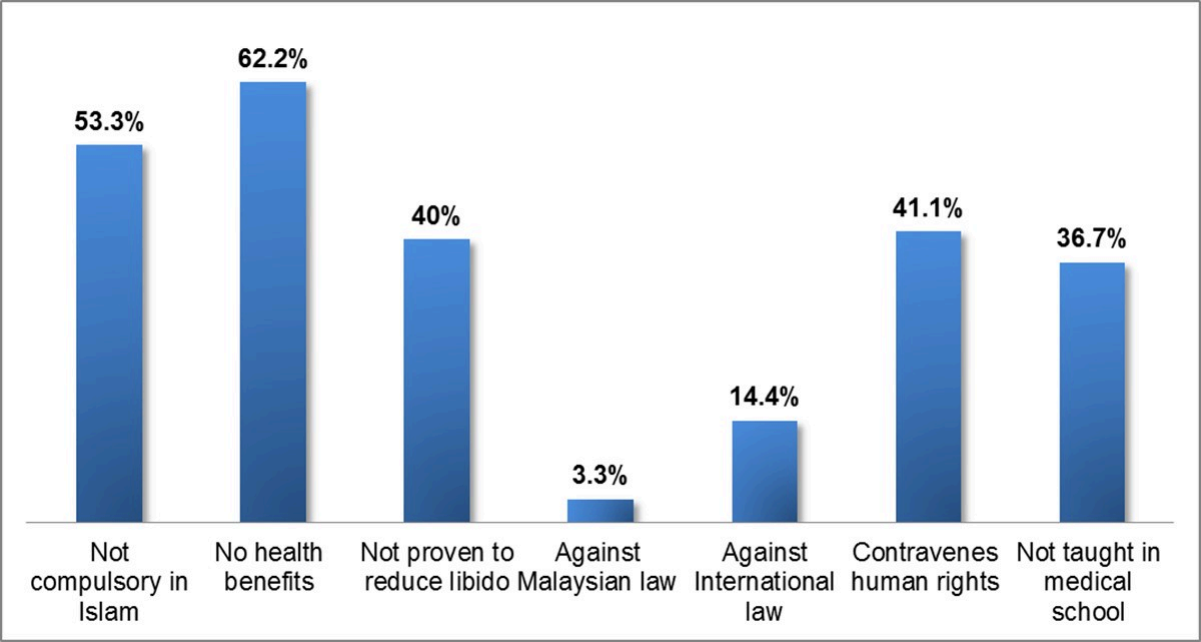
Reasons why FGC should not continue. FGC, female genital cutting.

Fig 2 shows the reasons why FGC should not continue: there are no health benefits (56), it is not compulsory in Islam (48), it contravenes human rights (37), it is not proven to reduce libido (36), it is not taught in medical school (33), it is against international law (13), and it is against Malaysian law (3).

### Regression analysis showing factors associated with conducting FGC

Table 7 shows the result of a binary logistic regression that was conducted to determine the significant factors associated with conducting FGC, which included age, sex, clinic ownership, knowledge about JAKIM fatwa, thinking that FGC mandatory in Islam, thinking that FGC is legal in Malaysia, thinking that FGC is encouraged in religion, thinking that FGC increases libido, and thinking that FGC should continue. The model had an overall correct predicted percentage of 88.5% and Nagelkerke R^2^ of 0.457. Being a woman (adjusted odds ratio [aOR] 4.4, 95% CI 1.9–10.0, *p <* 0.001), owning a clinic (aOR 30.7, 95% CI 12.0–78.4, *p <* 0.001) or jointly owning a clinic (aOR 7.61, 95% CI 3.2–18.1, *p <* 0.001), thinking that FGC is legal in Malaysia (aOR 2.09, 95% CI 1.02–4.3, *p* = 0.04), thinking that FGC is encouraged in religion (aOR 2.25, 95% CI 3.2–18.1, *p =* 0.04), and thinking that FGC should continue (aOR 3.54, 95% CI 1.25–10.04, *p =* 0.01) increased likelihood of practicing FGC.

**Table 7:** Regression analysis of factors associated with practising FGC.

	β	Standard error	Wald statistic	*P*	aOR (95% CI)
**Sex**					
Men (Ref)					
Women	1.482	0.420	12.461	<0.001	4.40 (1.93–10.02)
**Clinic**					
MoH (Ref)					
Self	3.424	0.479	51.097	<0.001	30.68 (12.0–78.44)
Joint	2.029	0.442	21.104	<0.001	7.61 (3.20–18.09)
**FGC legal in Malaysia**					
No (Ref)					
Yes	0.737	0.367	4.048	0.04	2.09 (1.02–4.29)
**Reason for doing it is that it is encouraged in religion**					
No (Ref)					
Yes	0.812	0.386	4.417	0.036	2.25 (1.06–4.81)
**FGC should continue**					
No (Ref)					
Yes	1.265	0.531	5.663	0.017	3.54 (1.25–10.04)

**Abbreviations:** aOR, adjusted odds ratio; FGC, female genital cutting; MoH, Ministry of Health

## Discussion

The findings of this study show that the prevalence of FGC practice among doctors in Malaysia was 20.5%. The practice was conducted by mostly female doctors who were trained by senior colleagues on girls less than 1 year of age in their clinics. Most doctors practiced type IV FGC, but there were a substantial number conducting type I. The reasons cited for the practice included harm reduction, religion and culture, and even cosmetic reasons was mentioned. Money, however, was not a motivating factor for the practice. Most doctors wanted the practice to continue.

On average, 26% of women have been cut by medical professionals; the rates vary between 1% and 74% among countries [16]. The 5 highest medicalization rates are reported in Egypt (38%), Sudan (67%), Guinea (15%), Kenya (15%), and Nigeria (13%), and the rates of medicalization are increasing [17]. In the current study, 20.5% of doctors who responded practiced FGC; however, due to grey areas concerning the legality of the practice, there is a possibility of underreporting, making the numbers reported conservative compared to the actual medicalization rate. This underreporting is also noted in Egypt [33], Nigeria [26], and Indonesia [34].

There is no official training on FGC in the medical curriculum. Like most other health practitioners who perform FGC elsewhere, such as in Nigeria, Egypt, and Indonesia [26, 27, 34, 35], the doctors in this study learned the skills from colleagues who themselves had no formal training. Unfortunately, parents who prefer their daughters be cut by healthcare professionals are unaware of the healthcare providers’ lack of knowledge and training related to FGC [32].

Most of the doctors in this study were female, just like reported by studies in Kenya [5] and Nigeria [33]. This could be because most mothers think it is a woman’s duty to perform FGC on girls [5] probably because FGC has been traditionally conducted by female midwives [32]. Unlike in parts of Africa where FGC is performed by medical personnel in homes or makeshift clinics [5], all FGC in the current study was conducted on girls of a very young age in the clinics owned or co-owned by the doctors—just like in other parts of South East Asia—to avoid embarrassment and the difficulty of restraining a bigger child [31, 36–38].

Unlike the traditional midwives in Malaysia, Thailand, Singapore, and Indonesia who practice type IV FGC [30–32, 34, 36, 38], a number of doctors in this study practiced more invasive forms of FGC by cutting parts of the clitoris (type I). Similar findings have been reported in Indonesia [34, 37]. Traditional practitioners usually tend to cut minimally for fear of bleeding and pain, but having anaesthetics and having an understanding of anatomy and physiology may result in doctors using deeper and more extensive cuts. And because the prepuce of the clitoris is small, there is a risk of injuring the clitoris or the surrounding area [20]. However, in some parts of Sudan, it is reported that medicalization has resulted in less severe forms of FGC [24].

The finding in this study that some doctors claimed harm reduction as their reason for practising FGC concurs with the findings of a review by Doucet and colleagues [12] and studies in Nigeria [26] and Egypt [27], where doctors practice FGC to prevent parents seeking traditional practitioners [16]. Religion and culture were motivations for the doctors in this study to conduct FGC just as in studies conducted in Nigeria [26, 35] and Egypt [33]. This finding also concurs with a review of literature by Doucet and colleagues [12] that found that FGC was justified for cultural reasons. Doctors who practiced FGC in this study were Malay Muslims who themselves were part of the community that they served, therefore some of them may have had the same religious, social, and cultural motivations as those who requested the service [27]. Some may have undergone FGC themselves or have maintained the tradition for their daughters [28]. Some doctors in this study cited cosmetics as a reason for doing FGC as found in studies in Egypt [27] and Indonesia [34]. Money was not the primary motivation to conduct FGC in the current study, as opposed to a literature review [12] and studies in Nigeria [26], Egypt [33], and Indonesia [39] that showed FGC to be a lucrative practice. In general, parents are not very concerned about the cost because they prefer and trust health providers and the formal health system [26].

Judging from the large number of doctors who wanted FGC to continue and their wish lists, it can be assumed that these doctors were unaware of the Sustainable Development Goal target 5.3 to eliminate all forms of FGC by 2030 [18] and the stand taken by the World Medical Association against doctors practising FGC [1]. A systematic literature review of health professionals’ knowledge, attitudes, and clinical practice toward FGC found that, although most doctors in the UK understood that FGC is illegal, the awareness of the UK FGC act ranged from 40% to 79%. In Belgium, only 45.5% of gynaecologists knew that FGC was illegal in the country. In the US, 56% of midwives knew that FGC was against the law, and less than half of Italian health professionals knew about the law prohibiting FGC in Italy. These figures, however, are higher than the 25% and 17% reported in Sudan and Egypt, respectively [40].

### Strengths and limitations

The main limitation of this study is the sampling. The sample size of the study was small, casting doubt on the representativeness of the sample. The representativeness of the sample (as opposed to its precision) is always an issue with survey research, and nonresponse may have influenced the results to an unknown extent and in unknown directions. The unfortunate problem is that this bias could only be measured by surveying the nonresponders. Low response rate is another limitation of the study, but considering the religious, cultural, and ethical sensitivities around the topic of FGC, a low response rate is not unexpected. The degree, and even the direction, of resulting bias can only be guessed at. We suggest future that research use survey methods more suited to sensitive issues such as respondent-driven sampling or snowball sampling [41], whereby the survey is propagated through networks of peers rather than directly administered. However, the strength of this study is that many interviews were conducted using snowball sampling, which helped in explaining some of the findings of this study. We recommend a large-scale study involving a bigger sample size and in-depth interviews among doctors who are from parts of Malaysia that this study did not include.

### Implications of the study

The information garnered by this study can be used to persuade MoH Malaysia and the Malaysian Medical Council to issue a statement against the practice. This will clarify the confusion of the doctors in Malaysia concerning the legality of the practice in the country. Fear of losing their medical licence may compel doctors to abide by the sanctions imposed. Because of the trust parents have toward doctors, they should be roped into the fight against FGC by training them on how to counsel parents who approach them for FGC. Having FGC integrated into the medical curriculum will help future doctors understand the ethical and legal position of the national and international medical community against the practice.

### Conclusion

There is a possibility that the prevalence of FGC reported in this study could be lower than the actual rate. The high rates of respondents who wanted the practice to continue is a cause of concern. The doctors in this study were beginning to practice type I FGC, which was unheard of among the traditional midwives, who only practiced type IV. It is imperative for MoH Malaysia and the MMC to take a clear stand against the medicalization of FGC.

## Supporting information

S1 STROBE Checklist STROBE, Strengthening the Reporting of Observational Studies in Epidemiology. (DOCX)

S1 Protocol Brief protocol of the FGC study. FGC, female genital cutting.
(DOCX)

S1 Questionnaire Questionnaire for the FGC study. FGC, female genital cutting.
(DOCX)

S1 Data Study data. (XLSX)

S1 Data Dictionary Data dictionary of the study data. (XLSX)

## Abbreviations

aOR: adjusted odds ratio
FGC: female genital cutting
FIGO: International Federation of Gynaecology and Obstetrics
MMC: Malaysian Medical Council
MoH: Ministry of Health
NGO: nongovernmental organisation
STROBE: Strengthening the Reporting of Observational Studies in Epidemiology
UN: United Nations
UNICEF: United Nations Children’s Fund
UNFPA: United Nations Population Fund
USD: US dollar
WHO: World Health Organization
