# Supplementary material for: Medicalization of female genital cutting in Malaysia: A mixed methods study
Source: PLoS Med. 2020 Oct 27;17(10):e1003303. doi: 10.1371/journal.pmed.1003303 (PMC7591112; doi:10.1371/journal.pmed.1003303)
Supplement: S1 Protocol — FGC, female genital cutting. (DOCX) [file pmed.1003303.s002.docx]

**Research Protocol**

**Title**

The Practice of Female Genital Cutting Among Medical Doctors in Malaysia

**Synopsis**

Female Genital cutting is now increasingly practiced by medical doctors. This trend of medicalization is a cause of concern and goes against every ethical code of conduct enshrined in the medical fraternity’s world over. At present nothing is known about the practice in Malaysia hence the proposed study to determine the practice and the factors which promote the practice among doctors.

**Abbreviations and acronyms**

FGC – Female Genital Cutting

FGM – Female Genital Mutilation

**Introduction**

The term Female Genital Mutilation/Cutting (FGM/C) refers to all procedures involving partial or total removal of the external female genitalia, or any other injury to the female genital organs for non-medical reasons. There are several types defined by the World Health Organization (WHO) [1] which varies within and between countries

**Type I:** Partial or total removal of the clitoris and/or the prepuce (clitoridectomy). Variation include type Ia - removal of the clitoral hood or prepuce only and type Ib - removal of the clitoris with the prepuce.

**Type II:** Partial or total removal of the clitoris and the labia minora, with or without excision of the labia majora (excision). Variations include, type IIa - removal of the labia minora only, type IIb - partial or total removal of the clitoris and labia minora, type IIc - partial or total removal of the clitoris, labia minora and labia majora.

**Type III:** Narrowing of the vaginal orifice with creation of a covering seal by cutting and apposition the labia minora and/or the labia majora, with or without excision of the clitoris (infibulation). Variations include type IIIa - removal and apposition of the labia minora, type IIIb - removal and apposition of the labia majora.

**Type IV:** Unclassified; all other harmful procedures to the female genitalia for nonmedical purposes, for example, pricking, piercing, incising, scraping and cauterization.

It has been estimated that worldwide more than 200 million girls and women have undergone some form of FGM/C [2], and approximately 3.6 million girls are cut each year. The decline in prevalence, despite decades of campaigning and even criminalizing FGM, is slow [3] probably because of the strong cultural and religious value and believe placed on this [4]. Although most of those who circumcise are traditional healers [5] who usually do not have any medical training and perform without any anaesthesia nor sterilization [6], more parents are choosing to have their daughters undergo the procedure by a health care provider preferably in a clinic to minimize pain and complications [7]. This trend known as medicalization of FGM/C is a serious global concern [8]. Medicalization are procedures performed by health care professionals or the use of medical instruments, antibiotics and/or anaesthetics by traditional practitioners also known as pseudo-medicalization [9]. The WHO defines Medicalization as the “situation in which FGM is practiced by any category of health-care provider, whether in a public or private clinic, at home, or elsewhere” [1]. It is estimated that worldwide, more than 18% of FGC is carried out by healthcare workers which include nurses, trained mid wives and other health care professionals, the rates vary between 1% and 74% between countries [10]. The involvement of health-care providers has been labelled as unprofessional and is a violation of the medical code of ethics and is illegal in some countries. Medicalization creates a false impression that the procedure is good for health or harmless and may create a sense of legitimacy for the practice [11]. There is also a concern that medicalization of the practice may develop into financial interest and could propagate the practice in both medicalized and unmedicalized form [10-12]. Most health-care providers who perform FGM/C are a part of the FGM/C practising community in which they serve and often have the same reasons as those requesting to conduct the procedure. Reasons given for the practice by the health care practitioner who conduct FGC are it is a medical indication, others consider it harmless, some perform the procedure because they support the patients socio cultural beliefs, others consider medicalization as a harm reduction and thus prevent the risks associated with FGC performed by traditional practitioners and by not providing the service the community will revert to traditional practitioners. However, there has been reports of financial gains as the motivation for the procedure [1, 10, 13, 14].

**Objectives**

To determine

the extent of medicalization in Malaysia and

the factors which influence the doctors to practice FGC.

**Methodology**

***Study design***: a mixed method (qualitative and quantitative) study design will be used among Muslim medical practitioners registered as members of major medical associations in Malaysia. ***Tools:*** a self-administered questionnaire will be used. The questionnaire along with a client information sheet will be posted to doctors who could reply using a stamped envelope with the investigators address. The questionnaire will have four sections, baseline information, practice, knowledge and future of FGC. For the qualitative part, in-depth interviews with the doctors who practice FGC will be conducted using a semi structured interview guide. ***Study population:*** Because FGC is only performed by Muslim doctors, the investigators will only enrol doctors from large medical associations in the country who have a big number of Muslim medical doctors as members. The associations will be asked to help distribute the questionnaires to their members. The association will be asked to identify those who are interested to participate in the in-depth interviews. There are no similar studies which have been conducted among medical doctors in Malaysia and there are no official statistics concerning the practice of FGC among doctors in Malaysia. However, the investigators believe a substantial number of doctors among the large population of Muslim doctors practice FGC. A sample size of 384 Muslim doctors will allow the study to determine the prevalence of those practising FGC with a confidence interval of ± 5%. For the qualitative part of the study, it is the intention of the investigators to interview the doctors until the data had reached saturation.

| Year | 2018 | | | | | | | | | | 2019 | | | | |
| --- | --- | --- | --- | --- | --- | --- | --- | --- | --- | --- | --- | --- | --- | --- | --- |
| Projects | mar | apr | may | jun | jul | aug | sep | oct | nov | dec | Jan - July | July - sept | Sept – Nov | Nov | DeC |
| Writing proposal | X | X |  |  |  |  |  |  |  |  |  |  |  |  |  |
| Obtaining an ethical approval |  | X | X |  |  |  |  |  |  |  |  |  |  |  |  |
| Grant application |  |  | X | X | X | X | X |  |  |  |  |  |  |  |  |
| Ground work |  |  |  |  |  |  |  | X | X | X |  |  |  |  |  |
| Data collection |  |  |  |  |  |  |  |  |  |  | X |  |  |  |  |
| Data analysis |  |  |  |  |  |  |  |  |  |  |  | X |  |  |  |
| Write up |  |  |  |  |  |  |  |  |  |  |  |  | X |  |  |
| Submission & Conferences |  |  |  |  |  |  |  |  |  |  |  |  |  | X | X |

**Data management**

All data related to the study will be kept in a cupboard in the principal investigators office which will be locked with access only available to the principle investigator. Every effort will be made to ensure the confidentiality of the participates.

**Statistical analysis**

Quantitative data will be analysed using SPSS version 18 and presented descriptively in tables and graphs. Prevalence of the practice of FGC will be reported. Chi square test was used to determine the factors which were significantly associated with those who practiced FGC. A binary logistics regression will be performed using independent factors which have a p value of 0.2 in the chi square test. Qualitative data will be collected using a semi structured questionnaire. Data will be collected until saturation of information is achieved. Saturation of data is considered to have achieved when no new information is availed from the respondents. The data will then be transcribed manually, organized, coded and recoded using N vivo. The clustered codes will be elicited as common themes by the investigators

**Ethical issues**

This study will be ethically conducted with all the participants required to provide an informed consent. The anonymity of the participants is assured, each participant will be assigned a unique code. A submission for ethical approval will be made to the Ritsumeikan Asia Pacific University Research Ethics Committee and a research grant will requested from the KAKENHI grant from Japan Society for the Promotion of Science.

**Dissemination of results**

Results of the study will be disseminated by way of publication and presentations at conferences and meetings

1. WHO: **Global strategy to stop health-care providers from performing female genital mutilation**. In*.* Geneva, Switzerland World Health Organization; 2010.

2. UNICEF: **Female Genital Mutilation/Cutting: What might the future hold?** In*.* New York: Female Genital Mutilation/Cutting: What might the future hold?,; 2014: 6.

3. Johansen REB, Diop NJ, Laverack G, Leye E: **What Works and What Does Not: A Discussion of Popular Approaches for the Abandonment of Female Genital Mutilation**. *Obstetrics and Gynecology International* 2013, **2013**:10.

4. Kontoyannis M, Katsetos C: **Female gentital mutilation**. *Health Science Journal* 2010, **4**(1):31-36.

5. UNICEF: **Female Genital Mutilation/Cutting: A statistical overview and exploration of the dynamics of change**. In*.* New York, United States of America: Statistics and Monitoring Section,Division of Policy and Strategy; 2013.

6. Momoh C: **Female genital mutilation**. *Trends in Urology, Gynaecology & Sexual Health* 2010, **15**(3):11-14.

7. El-Gibaly O, Ibrahim B, Mensch BS, Clark WH: **The decline of female circumcision in Egypt: evidence and interpretation**. *Soc Sci Med* 2002, **54**(2):205-220.

8. UNFPA: **Brief on the medicalization of female genital mutilation**. In*.* Edited by UNFPA, UNICEF, WHO: UNDF; 2018: 1-2.

9. Kimani S, Shell-Duncan B: **Medicalized Female Genital Mutilation/Cutting: Contentious Practices and Persistent Debates**. *Curr Sex Health Rep* 2018, **10**(1):25-34.

10. Pearce AJ, Bewley S: **Medicalization of female genital mutilation. Harm reduction or unethical?** *Obstetrics, Gynaecology and Reproductive Medicine* 2014, **24**(1):29-30.

11. El-Gibaly O, Aziz M, Abou Hussein S: **Health care providers’ and mothers’ perceptions about the medicalization of female genital mutilation or cutting in Egypt: a cross-sectional qualitative study**. *BMC International Health and Human Rights* 2019, **19**(1):26.

12. Doucet M-H, Pallitto C, Groleau D: **Understanding the motivations of health-care providers in performing female genital mutilation: an integrative review of the literature**. *Reproductive health* 2017, **14**(1):46-46.

13. Johansen RE: **Health professionals should never perform female genital mutilation**. Switzerland: Institut international des Droits de l'Enfant; 2011.

14. Obianwu O, A. A, O. D: **Understanding medicalisation of Female Genital Mutilation/Cutting (FGM/C): a qualitative study of parents and health workers in Nigeria**. In: *Evidence to End FGM/C: Research to Help Women Thrive.* New York: New York: Population Council; 2018.
