# Supplementary material for: Medicalization of female genital cutting in Malaysia: A mixed methods study
Source: PLoS Med. 2020 Oct 27;17(10):e1003303. doi: 10.1371/journal.pmed.1003303 (PMC7591112; doi:10.1371/journal.pmed.1003303)
Supplement: S1 Questionnaire — FGC, female genital cutting. (DOCX) [file pmed.1003303.s003.docx]

**TITLE: MEDICALIZATION OF FEMALE GENITAL CUTTING IN MALAYSIA**

**Date: ______/_______/______ Code:__________________**

***Please circle/tick the answers where appropriate***

**A. Demography**

1. **Age: 2. Gender: Men / Women**
2. **Basic medical degree (Country): 4. Year graduate:**
3. **Post graduate degree: 6. Country:**

**B. Clinic**

1. **Clinic ownership: Self / Joint / MOH**
2. **Years working in present clinic: ________________ year/s**
3. **Clinic location - State (e.g. Kedah): _____________________________**
4. **Clinic location – Rural / Urban**

**C. FC Practice:**

1. **Do you practice FGC: Yes / No**

***(if yes, please proceed to question 3)***

1. **If you do not perform FGC, why?**

**It is against the law / It is against your beliefs / It is against Islam / You have no training**

***(please proceed to section D)***

1. **How long have you been practicing FGC (in years) : _________________**
2. **Did you receive any training on FGC? : Yes / No**

***(if no, please proceed to question 6)***

1. **If yes, where did you receive your training on FGC:**

**Medical School / Online / Colleagues / Self-taught / Religious personnel / Mak Bidan (Traditional midwives)**

1. **Approximately how many FGC’s in average do you conduct in a month?_________________________________**
2. **Do you use any local anaesthesia when you perform FGC? Yes / No**
3. **Is there any bleeding during the procedure? Yes / No**

**(*if no, please proceed to question 10)***

1. **If yes, how much blood?**

**A drop / Gauze full / More than a gauze full**

1. **Have you encountered any complications when performing FGC? Yes / No**

***(if no, please proceed to question 12)***

1. **If Yes what? ___________________________________________________**
2. **Do you screen the patients before conducting FGC? Yes / No**

***(if no, please proceed to question 15 )***

1. **If yes, for : Infectious diseases / Bleeding disorders / Others**
2. **If yes, by : History / Blood tests / others __________________**
3. **Please highlight the area FGC (*picture*) is done and tick what is done (*box*)**

**
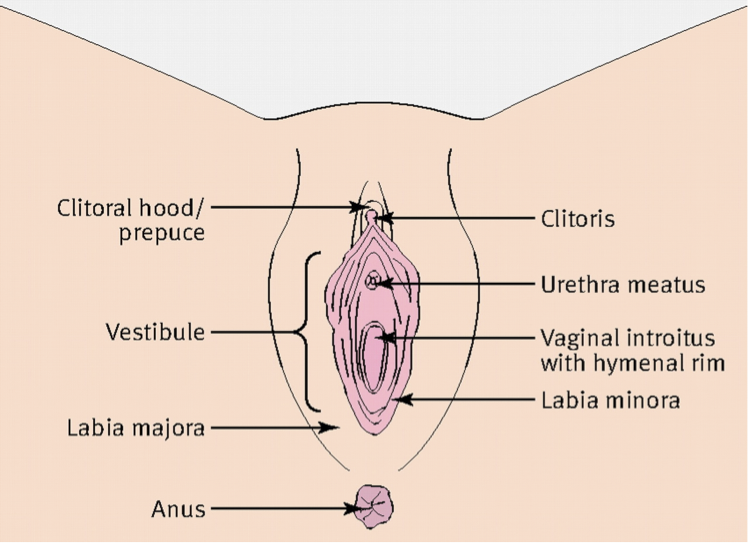
**

**Excision of the prepuce**

**Prick the prepuce**

**Nick the prepuce**

**Nick the tips of clitoris**

**Prick the clitoris**

**Other:___________________________**

1. **What is done: Cut / Teased out / Pricked / Rubbed**
2. **Which instrument is used?**

**Scissors / Surgical blade / Surgical needle / Other non-medical equipment ____________________________________**

1. **What is applied post procedure?**

**Antiseptic / Antibiotic ointment / Nothing / Other___________________________________**

1. **What is the most common age you perform the procedure? ________________**

1. **What age do you suggest the procedure should be performed?____________________**
2. **What is the average charge for FGC? RM_____________**
3. **Why do you perform FGC?**

| **Reasons** | **Multiple Choice- please tick** |
| --- | --- |
| **Religion** |  |
| **Health reasons** |  |
| **Hygiene** |  |
| **Reduce libido** |  |
| **Increase sensitivity/libido** |  |
| **Help during childbirth** |  |
| **Peer pressure** |  |
| **Family pressure** |  |
| **Monetary reasons** |  |
| **Others (specify)** |  |

1. **Is consent obtained? Verbal / Written / None**

**D. Knowledge**

**1. Do you know the 2009 Fatwa by JAKIM (National Council on Islamic Religious Affairs) declared female circumcision to be obligatory (*wajib*) for all Muslim women? Yes / No**

**2. Is female circumcision mandatory according to Islam Yes / No / Don’t know**

**3. Is female circumcision legal in Malaysia? Yes / No / Don’t know**

**4. Is female circumcision conducted among all Muslim sects in the world? Yes / No**

***If you agree to the practice of FC please continue otherwise proceed to section E***

**5. What is the ideal age, a girl should be circumcised? ____________ months**

**6. What is/are the reason/s for doing female circumcision in Malaysia? (*Multiple response*)**

| **Reasons** | **Multiple Choice- please tick** |
| --- | --- |
| **Religion –compulsory** |  |
| **Religion –encouraged but not compulsory** |  |
| **Health reasons** |  |
| **Hygiene** |  |
| **Reduce libido** |  |
| **Increase sensitivity/libido** |  |
| **Helps during childbirth** |  |
| **Peer pressure** |  |
| **Family pressure** |  |
| **Others (specify)** |  |

**6. Who performs female circumcision in Malaysia? (*Multiple response*)**

| **Reasons** | **Multiple Choice – Please tick** |
| --- | --- |
| **Traditional mid wives** |  |
| **Trained mid wives from KKM** |  |
| **Nurses** |  |
| **Medical doctors** |  |
| **Medical specialists** |  |

**7. Who do you think should perform female circumcision? (*single best answer*)**

| **Reasons** | **Single Choice – please tick** |
| --- | --- |
| **Traditional mid wives** |  |
| **Trained mid wives from KKM** |  |
| **Nurses** |  |
| **Medical doctors** |  |
| **Medical specialists** |  |

**8. Why do you feel FC should be performed in clinics (*multiple choice*)**

|  | **Multiple Choice – Please tick** |
| --- | --- |
| **No complications** |  |
| **Less complications** |  |
| **Hygienic** |  |
| **To comply with traditions & culture** |  |
| **Safe** |  |
| **Experience** |  |
| **Expertise** |  |

**E. Future**

1. **Do you think the practice should continue? Yes / No**

***(if yes, proceed to question 3)***

1. **Why FGC should not continue?**

| **Reasons** | **Multiple Choice- please tick** |
| --- | --- |
| **It is not compulsory in Islamic religion** |  |
| **It does not bring about any health benefits** |  |
| **It is not proven to reduce libido** |  |
| **It is against Malaysian law** |  |
| **It is against international law** |  |
| **It is contravenes human rights** |  |
| **It is not taught in medical schools** |  |

1. **Your suggestions to continue this practice**

| **Reasons** | **Multiple choice- please tick** |
| --- | --- |
| **Have official training in medical schools** |  |
| **Have religious experts define the confines of the practice** |  |
| **Have regular updates on the practice** |  |
| **Laws enacted to make the practice legal** |  |
| **MMC to officially declare the practice legal** |  |

***Thank you for participating in this study***
